# Transactions of the Buffalo Obstetrical Society

**Published:** 1885-06

**Authors:** 


### Transactions of the Buffalo Obstetrical Society.

*Stated Meeting, April 28, 1885.*

The Vice-President, Dr. R. L. Banta, in the Chair; Dr. W. D. Greene, Secretary *pro tem.*

Dr. H. D. Ingraham read a paper on "Retro-Displacements of the Uterus; their Etiology and Pathology." He embraced, under this title, the conditions known as retroversion and retroflexion, neither of which conditions often existed alone, but each was more or less complicated by the other.

The causes of backward displacements of the uterus might be due to inflammatory conditions existing in the pelvic cavity; to non-inflammatory pelvic faults; to conditions of the adjoining parts; or the causes might reside in the general system. Errors in development were also among the causes of versions and flexions of the womb; and this condition was especially liable to happen at puberty, when anything which retarded, prevented, or arrested nutrition, such as poor food, bad air, malassimilation, insufficient exercise, and the moral and social errors which tend to sexual precocity, were such action factors in the production of innutrition and faulty developmental growth. But the most frequent cause of retro-displacements was to be found in defective involution of the uterus, following childbirth and abortion. While the recently delivered uterus was still within the abdominal cavity, the vertebral column prevented its falling backwards; but when it sank below the promontory of the sacrum, and became subjected, while its ligaments were relaxed, to the pressure of the superincumbent viscera, retro-displacements were quite likely to be produced.

In injuries of the cervix, during parturition, the circulation became impeded and irritative hyperæmia resulted, often causing retro-displacements. The relaxed condition of the uterus, during the puerperal convalescence, gave rise to a softened condition of the parenchyma, and hence many mechanical causes operated in the production of versions and flexions, such as prolonged dorsal decubitus with a tightly applied binder, and the like.

The early application of the child to the breast was an important factor in securing involution, hence these mal-positions were not as common in women who nursed their children. Menstruation coming on prematurely and suspending lactation, arrested involution and thereby served to produce versions and flexions. All inflammatory conditions of the uterus that followed labor or abortion, aside from the mere arrest or retardation of involution, were important factors in the production of retro-displacements, softening as the result of acute inflammation being the most common of all.

Congestive dysmenorrhœa, menorrhagia, and metorrhagia, all tended to cause softening of the tissues, and hence were factors in the production of versions and flexions; so, too, was atrophy of the uterine muscular substance. Morbid growths, which became developed in the uterine walls, thus causing flexions and versions, were numerous, the most common being the so-called fibroid tumor. Those retro-displacements of the uterus due to inflammatory conditions existing in the pelvic cavity, might be caused by adhesions of the uterus to the adjacent parts. Septic poisoning, gonorrhœal infections, taking cold at the menstrual period, instrumental deliveries, and other like causes, might produce inflammation of the pelvic peritoneum, or the cellular tissue, and result in retro-displacements. An abscess, the too free use of caustics, or a fistulous opening, might cause cicatricial contractions of the tissue behind the cervix, and thus a backward displacement.

Of the many non-inflammatory conditions of the pelvis which assist in the production of retro-displacements, the most common was, perhaps, the lessened curve of the lumbar vertebræ, which took away just so much support from the abdominal viscera, and thus allowed them to gravitate within the pelvic cavity. Pelvic tumors, extra-uterine pregnancy, and peri-uterine hematocele, might act as displacing forces, and, in some cases, produce retro-displacements. Over-distension of the bladder, habitual constipation, and straining at stool, tended to provoke these displacements. Perineal ruptures, causing impairment of the integrity of the supporting column, are frequent sources of uterine displacements. He laid great stress upon the fact that constipation and ruptures of the perineum were more frequent causes of uterine mal-positions than was generally supposed.

Many general and special causes operated to produce these mal-positions of the uterus, among which he cited occupation; the constant use of the sewing machine, and prolonged standing on the feet, as girls in stores and shops are obliged to do; masturbation, to a certain extent; frequent child-bearing, prolonged lactation, various anti-conception measures, and ill-assorted marriages; and, in fact, anything which lowered the general tone of the system, came with this category.

The pathology of uterine retro-displacements had a very important bearing upon their treatment. In that form in which the mal-position developed as the organ developed, there was usually no particular pathological change in its texture. In that class in which the uterus was once in a normal position and properly developed, we might find changes in the muscular tissue of the uterus, in the endometrium, in the cavity itself, in the blood-vessels, and, in fact, in any portion of the uterine structures. These changes might also produce alterations in the subjacent parts. In most cases of acquired flexion, the uterine tissue became softened and relaxed, as well as hypertrophied. The softened and relaxed condition was usually more marked at the internal os than elsewhere, and it was there that the displacing forces exerted their greatest strain. In flexions following abortion or parturition, the uterus was found enlarged, its walls softened, and the mucous membrane denuded, while involution of the organ was in abeyance. In such cases the version or flexion was usually due to loss of tone in the uterine tissue, and not to pressure of the superincumbent organs. Depending upon the length of time the displacement has existed, the walls of the uterus were either stiff, thick and resistant, or elastic, yielding, loose, and the vessels turgid with blood. In chronic flexion there was often a marked induration, without atrophy, in which condition the resistance to replacement was great, as the uterus quickly returned to its dislocated position upon withdrawal of the replacing force.

The external os, in flexions, was usually patulous, and often in a state of granular degeneration. The state of the cavity was generally catarrhal, due either to pressure at the internal os, which prevented escape of the normal secretions; or to passive hyperæmia, caused by distortion of the uterine connection with neighboring parts, producing hypersecretion; in either case there was an expansion of the cavity. Traces of inflammation of the mucous membrane, both recent and chronic, were frequently found, such as red, rounded or flattened granulations, which obstruct the os internum; and occasionally granular swelling of the mucous membrane could be seen through the cervical canal. These morbid conditions added to the contraction of the canal, sometimes rendering it nearly impervious, and greatly impeding the escape of the menstrual discharges. In all retro-displacements the circulation was abnormal. If there was not active, then there was usually passive congestion or inflammation of the parts. Relatively there was more blood in the uterus than in the healthy state, the arteries carrying the usual quantity to the organ, but the veins being distorted, compressible, and valveless, were unable to return it as freely as required to maintain the equilibrium.

*Discussion*—Dr. J. W. Keene called attention to the fact that the causes of retroflexion were attributed, by very many text books, to the habitual dorsal decubitus during puerperal convalescence. He thought the profession was all at sea on the question of uterine displacements, as indicated by the great diversity of opinions on the subject. He was inclined to believe that the attachment of the placenta to the anterior wall of the uterus, with the consequent thickening at the site, would have a tendency to prevent retroflexion.

Dr. F. H. Potter said that he had been taught that the puerperal binder was liable to produce, among other things, uterine displacements; consequently, he was not favorable to its use to the extent that it was generally employed.

Dr. P. W. Van Peyma, in referring to the causes of the condition under discussion, selected two cases of backward displacement, produced by jumping, but which were easily restored.

Dr. George E. Fell thought it strange that the uterus was so often displaced, when other organs of the body, subjected to similar influences, escaped.

The Vice-President said that although the treatment of these displacements was to be discussed at some future time, when they could be seen early they were much more tractable. The difficulty was that they rarely presented themselves to the observation of physicians until some dire necessity occurred, when the mischief done was already so great that it was often difficult to restore the organ to its normal state. He believed the moral effect of treatment was beneficial, in many cases, where the displacement was more imaginary than real.

Dr. W. D. Greene was a believer in the abdominal bandage for the first few hours after delivery, or until the danger of post partum hemorrhage had practically passed. If the woman was told to lie on either side the greater part of the time, he did not see how the bandage could do harm if properly adjusted.

Dr. Ingraham closed the discussion by saying that it was formerly his custom to use the bandage after delivery; that he discarded it altogether at one time; but that he had again commenced its use. He always requested the patient to lie upon her stomach a good part of the time, for the first few days following parturition, and, when this plan was followed, a good getting up was usually the result. He advocated the practice of always making an examination of the gentalia before finally dismissing a parturient patient, with a view to ascertain the precise condition of the parts, in order that intelligent advice may be given, should there be found any rents or other abnormal conditions.

